# Discovery of immunodominant T-cell epitopes reveals penton protein as a second immunodominant target in human adenovirus infection

**DOI:** 10.1186/s12967-016-1042-2

**Published:** 2016-10-07

**Authors:** Sabine Tischer, René Geyeregger, Julian Kwoczek, Albert Heim, Constanca Figueiredo, Rainer Blasczyk, Britta Maecker-Kolhoff, Britta Eiz-Vesper

**Affiliations:** 1 Institute for Transfusion Medicine, Hannover Medical School, Carl-Neuberg-Strasse 1, 30625 Hannover, Germany; 2 Integrated Research and Treatment Center (IFB-Tx), Hannover Medical School, Carl-Neuberg-Strasse 1, 30625 Hannover, Germany; 3 St. Anna Kinderkrebsforschung e.V., Children’s Cancer Research Institute, Vienna, Austria; 4 Institute for Virology, Hannover Medical School, Carl-Neuberg-Strasse 1, 30625 Hannover, Germany; 5 Department of Paediatric Haematology and Oncology, Hannover Medical School, Carl-Neuberg-Strasse 1, 30625 Hannover, Germany

**Keywords:** Human adenovirus, Penton, T-cell epitope, T-cell monitoring, Immunotherapy

## Abstract

**Background:**

Human adenovirus (HAdV) infections remain a significant cause of morbidity and mortality after hematopoietic stem cell transplantation (HSCT). Efficient antiviral T-cell responses are necessary to clear infection, which is hampered by delayed immune reconstitution and medical immunosuppression after HSCT. Protective immunity may be conferred by adoptive transfer of HAdV-specific T cells. For identification of patients at risk and monitoring of treatment responses diligent assessment of anti-HAdV cellular immune responses is crucial. The HAdV-derived protein hexon has been recognized as a major immunodominant target across HAdV species. We aimed at identifying further targets of protective anti-HAdV immune response and characterizing immunogenic epitopes.

**Methods:**

Nineteen candidate nonamers from hexon and penton proteins were identified by epitope binding prediction. Peptides were synthesized and tested for in vivo immunogenicity by screening peripheral blood mononuclear cells from healthy volunteers (n = 64) and HAdV-infected stem cell recipients (n = 26) for memory T cells recognizing the candidate epitopes in the context of most common HLA alleles.

**Results:**

Functional CD8^+^ T cells recognizing seven epitopes were identified, among them four penton-derived and two hexon-derived peptides. The HLA-A*01-restricted penton-derived peptide STDVASLNY (A01Penton_STDV_) and HLA-A*02-restricted hexon-derived peptide TLLYVLFEV (A02Hexon_TLLY_) were recognized by more than half of the persons carrying the respective HLA-type.

**Conclusions:**

Thus, the HAdV-derived penton protein is a novel major target of the anti-HAdV immune response. Identification of new immunodominant epitopes will facilitate and broaden immune assessment strategies to identify patients suitable for T-cell transfer. Knowledge of additional target structures may increase T-cell recovery in manufacturing processes.

**Electronic supplementary material:**

The online version of this article (doi:10.1186/s12967-016-1042-2) contains supplementary material, which is available to authorized users.

## Background

Human adenovirus (HAdV) infection constitutes a major cause of morbidity and mortality in patients undergoing allogeneic hematopoietic stem cell transplantation (HSCT). The incidence of HAdV infection ranges from 5 to 30 %, with pediatric recipients showing the highest rates of infection with up to 83 % lethality [1–6]. Monitoring for HAdV infection and therapeutic intervention (reduction of immunosuppression, antiviral treatment) may reduce mortality due to HAdV in pediatric HSCT recipients [7]. However, antiviral treatments for HAdV infection with agents like cidofovir and ribavirin are associated with toxicity and may result in delayed immune reconstitution. Previous studies clearly indicate that T cells, the most potent effectors of the human immune system, are crucial for HAdV clearance [2]. It was demonstrated that children with HAdV-associated mortality had no HAdV-specific T cells, whereas patients who cleared HAdV infection showed HAdV-specific T-cell responses [2, 8]. Adoptive transfer of HAdV-specific T cells offers an effective and non-toxic immunotherapeutic strategy to reduce or prevent the clinical manifestation of HAdV in HSCT recipients with no or low numbers of HAdV-specific T cells [2, 8–12]. Monitoring HAdV-specific T-cell immunity may improve risk assessment in HSCT recipients and enhance treatment efficacy by determining the optimal time point for adoptive T-cell transfer. The median time between the first detection of HAdV DNA in the blood and the onset of symptoms is 3 weeks, which therefore seems to be the optimal time point for adoptive T-cell transfer [2, 13, 14]. Since the generation of short-term in vitro generated virus-specific T-cell lines takes about 3 weeks including quality controls, the production should start even earlier at the time of high viral load in stool (>10^6^ copies) [12, 15].

The 70 different human HAdV types identified to date are divided into seven species (A to G) [16, 17]. Type 31 (of species HAdV-A) and HAdV 1, 2, and 5 (of species HAdV-C) are the most prevalent types in HSCT recipients [4–7]. Occasionally, types of species HAdV-B can be observed in adult HSCT recipients [18]. The major capsid protein hexon serves as an immunodominant target antigen across the different HAdV types, but few hexon-derived epitopes have been identified as immunodominant so far [13, 19–23]. Most of these epitopes are highly conserved, demonstrating that HAdV-specific T cells can cross-react across HAdV species and may therefore provide protection against a wide range of HAdV types [20]. HAdV-specific T-cell responses to the recombinant hexon protein, the overlapping peptide pool covering the complete hexon sequence, HLA-restricted peptides, and whole viral lysates have been investigated. A study by Feuchtinger et al. revealed that 10.5 % of donors had a specific T-cell response to the whole adenovirus but no response to the hexon protein, while 17 % of donors had no detectable T-cell response to HAdV [11]. Moreover, Zandvliet et al. detected specific CD8^+^ T cells in 6/16 healthy donors (37.5 %) after stimulation with the 15-mer hexon peptide pool, but only 3/16 donors (18.8 %) had specific T cells for known CD8^+^ hexon epitopes [24]. Sukdolak et al. observed a specific T-cell response to the 15-mer hexon peptide pool in 73 % of HAdV seropositive healthy donors, while 30 % were classified as high responders and 43 % as low responders [25]. Interestingly, 27 % of all HAdV seropositive healthy donors tested showed no response to the hexon peptide pool. These results underline the need to identify more immunogenic T-cell epitopes to improve the selection of HAdV-specific T cells for adoptive transfer and the immunomonitoring of high-risk patients.

T-cell epitopes can be identified by direct or reverse immunology. Various computer algorithms have been developed over the past years that allow for the prediction of peptide binding to MHC class I and II molecules, proteasome cleavage patterns and transporter associated with antigen processing translocation [26]. Naturally presented CD8^+^ T-cell epitopes are usually among the top-scoring 2 in 80 % of all predictions, whereas the reliability of CD4^+^ T-cell epitope prediction is much lower due to the more variable pocket binding behavior of MHC class II molecules [27]. SYFPEITHI [26, 28, 29], BIMAS [26, 30] and NetChop [31] are the most widely used algorithms to identify cytotoxic T lymphocyte (CTL) epitopes in viral, microbial, and tumor antigens. These well-established algorithms, which have been validated and compared [26], were employed in this study to predict new HAdV epitopes.

The major focus of this study was to identify and evaluate novel immunodominant HAdV-specific T-cell epitopes by analyzing the main structural proteins, hexon and penton. HLA-A*01-, A*02-, A*03- and B*08-restricted peptide epitopes within conserved protein regions (Table 1) were pre-selected based on the predictions of several established computer algorithms. Immunogenicity of the top-ranked epitopes was investigated by established methods: IFN-γ-based EliSpot, cytokine secretion assay (CSA), peptide MHC (pMHC) multimer staining and multicolor flow cytometry. Four of the selected peptide candidates were classified as low immunodominant and two as high immunodominant according to the number of responders in the healthy donors and HAdV-infected HSCT recipients. This paper describes for the first time the immunogenic potential of penton-derived epitopes and demonstrates that the penton, as an immunological target, it is not secondary to the hexon. Expanding the repertoire of immunodominant HAdV-specific T-cell epitopes will enable more precise immunomonitoring and more effective multi-epitope-based T-cell therapy by targeting epitopes presented in a broader array of HLA molecules.

**Table 1:** Predicted peptide candidates used for HAdV-specific T-cell screening in healthy donors

HLA class I restriction	Sequence [aa]	Abbreviation	HAdV species: type [cross-reactivity]	Responders [EliSpot]	T-cell response [reference]
HLA-A*01:01	TDLGQNLLY	A01Hexon_TDLG_	C: 1, 2, 5	13/18	High [13, 20, 21]
HLA-A*01:01	TNDQSFNDY	A01Hexon_TNDQ_	A: 31, B: 3, C: 1, 2, 5	2/12	No [21]
HLA-A*01:01	QNDPTVVMY	A01Hexon_QNDP_	A: 31	0/8	NA
HLA-A*02:01	TLLYVLFEV	*A02Hexon*_*TLLY*_	A: 31, C:1, 2, 5	28/41	High [13, 22, 23]
HLA-A*02:01	TLAVGDNRV	A02Hexon_TLAV_	A: 31, B: 3, C: 1, 2, 5	0/5	No [13]
HLA-B*08:01	GLRYRSMLL	B08Hexon_GLRY_	A: 31, B: 3, C: 1, 2, 5	0/5	Minimal [20]
HLA-B*08:01	DLQDRNTEL	*B08Hexon*_*DLQD*_	A: 31, B: 3, C: 1, 2, 5	6/27	NA
HLA-A*01:01	STDVASLNY	*A01Penton*_*STDV*_	C: 1, 2, 5	25/38	NA
HLA-A*01:01	SNDSTFTQY	A01Penton_SNDS_	C: 1, 2, 5	0/8	NA
HLA-A*01:01	SSDIASLNY	A01Penton_SSDI_	A: 31	0/5	NA
HLA-A*01:01	LTDHGTLPL	A01Penton_LTDH_	A: 31, B: 3, C: 1, 2, 5	0/5	NA
HLA-A*02:01	ILHTNMPNV	*A02Penton*_*ILHT*_	A: 31, C: 1, 2, 5	7/28	NA
HLA-A*02:01	ALGIVSPRV	A02Penton_ALGI_	A: 31, C: 1, 2, 5	0/6	NA
HLA-A*02:01	GNIPALLDV	A02Penton_GNIP_	A: 31, B: 3, C: 1, 2, 5	0/6	NA
HLA-A*03:01	VLESDIGVK	A03Penton_VLES_	A: 31, B: 3, C: 1, 2, 5	0/6	NA
HLA-A*03:01	LLPGCGVDF	A03Penton_LLPG_	A: 31, B: 3, C: 1, 2, 5	0/6	NA
HLA-B*08:01	NTKYRSWYL	B08Penton_NTKY_	A: 31	0/7	NA
HLA-B*08:01	DSKGRSYNL	*B08Penton*_*DSKG*_	A: 31	9/36	NA
HLA-B*08:01	LTKDKQVEL	B08Penton_LTKD_	C: 1, 2	0/5	NA
HLA-B*08:01	DSKKRSYNL	*B08Penton*_*DSKK*_	C: 1, 2, 5	15/36	NA

Names, sequences and abbreviations of 19 epitope candidates (plus one reference epitope, A01Hexon_TDLG_) predicted for frequent HLA class I alleles and clinically relevant HAdV types. The immunogenicity of epitope candidates was evaluated by pre-screening HAdV-specific T-cell responses to the 19 synthesized peptides in healthy donors using IFN-γ EliSpot assay. Seven of 19 peptide candidates induced HAdV-specific T-cell responses, six of which (highlighted in italic) were classified as immunodominant. The following epitope sequences were published elsewhere: A01Hexon_TDLG_ [13, 20, 21], A01Hexon_TNDQ_ [21], A02Hexon_TLLY_ [13, 22, 23], A02Hexon_TLAV_ [13], and B08Hexon_GLRY_ [20] (*NA* not applicable)

## Methods

### Study population

The current study has been approved by the Internal Review Board of Hannover Medical School. Following written informed consent peripheral blood was obtained from 64 healthy platelet donors from the Hannover Medical School (MHH) Institute for Transfusion Medicine and 26 pediatric patients after HSCT with detectable HAdV-DNA in blood and/or stool. Healthy donors had no prior history of blood transfusion and no signs of acute infection. All donors and patients were typed for HLA class I and class II alleles at the four-digit level by sequence-based typing [32]. Informed consent was obtained from all donors and patients as approved by the Ethics Committee of Hannover Medical School, and trial subject data were treated as confidential information protected by medical confidentiality.

### Epitope prediction

**Fig. 1:**
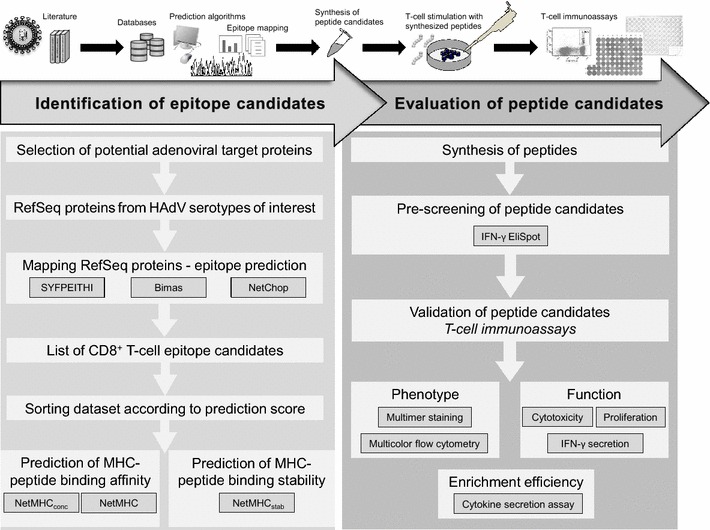
Schematic overview of the experimental approach for the identification and evaluation of novel CD8^+^ T-cell epitopes. The identification and evaluation of epitope candidates were the two major steps of the general workflow. First, epitope candidates were mapped by reverse immunology using different prediction algorithms for peptide binding affinity and stability. Second, the highest-scoring peptide candidates were synthesized and evaluated for immunogenicity by T-cell pre-screening and T-cell immunoassay

HAdV hexon and penton protein sequences restricted to the types 1, 2, 3, 5, and 31 were obtained from the SwissProt database (http://www.uniprot.org). Epitope prediction programs SYFPEITHI (http://www.syfpeithi.de) [28, 29], BIMAS (http://www.bimas.cit.nih.gov) [30], and NetChop (http://www.cbs.dtu.dk) [31] were used to predict nonamers capable of binding to HLA- A*01:01, A*02:01, A*03:01 and B*08:01 molecules (Fig. 1). Epitope candidates were only selected if identified by all programs according to their predictive scores (Table 1). The N_ET_MHC_STAB_ [33], N_ET_MHC, and N_ET_MHC_cons_ (Fig. 1) prediction algorithms provided by the Center for Biological Sequence Analysis (CBS, http://www.cbs.dtu.dk) was used to predict the stability of pMHC complexes for all database-available HLA types.

### Synthetic peptides and peptide pools

Peptides of the 19 top-scoring epitope candidates (Table 1) were synthesized (China Peptides, Shanghai, China; ProImmune, Oxford, UK) and used for pre-screening and T-cell immunoassays (Fig. 1). The overlapping peptide pools of hexon (HAdV5Hexon_pp_, Miltenyi Biotec, Bergisch Gladbach) and penton (HAdV5Penton_pp_, Miltenyi Biotec) were further used as stimuli of antiviral memory T cells. The HLA-A*01-restricted hexon-derived peptide TDLGQNLLY (A01Hexon_TDLG_, ProImmune, Table 1) was used as a positive control. Peptide binding assays were performed using two additional HLA-restricted peptides from phosphoprotein 65 (pp65) of the human cytomegalovirus (YSEHPTFTSQY: A01pp65_YSEH_ and NLVPMVATV: A02pp65_NLVP_, ProImmune) as positive controls.

### HLA class I peptide binding assay

The T2 peptide binding assay was performed with the HLA-A*01- and HLA-A*-02-restricted peptide candidates as described previously [34]. To determine peptide binding to HLA-A*01:01 molecules, T2 cells were transfected to express membrane-bound HLA-A*01:01 [35]. Briefly, 1 × 10^6^ T2 cells/ml were pulsed with 50 μg/ml peptide (Table 1) and 5 μg/ml beta-2 microglobulin (β2 m, Sigma, St Louis, MO, US) in serum-free medium for 15–18 h at 37 °C. T2 cells incubated without peptide served as controls. HLA expression levels were determined by flow cytometry (FACSCanto II and FACSDiva V6.1.2 software, BD Biosciences, San Jose, CA) using the monoclonal antibodies (mAb) HLA-ABC fluorescein (FITC, w6/32, AbD Serotec, Ltd-Kidlington, UK) and anti-HLA-A*02 phycoerythrin (PE, BB7.2, Biolegend, San Diego, CA, US).

### Screening for HAdV peptide-specific HLA-restricted T cells

The IFN-γ EliSpot assay was used for peptide screening to enumerate HAdV-specific IFN-γ-producing T cells [25, 36]. Briefly, peripheral blood mononuclear cells (PBMCs) were plated at a density of 2.5 × 10^5^ cells/well in triplicate wells and incubated overnight in the presence of the investigated peptides (10 µg/ml) and peptide pools (1 µg per peptide/ml). PBMCs cultured with medium alone or in the presence of 1 µg/ml staphylococcal enterotoxin B (SEB, Sigma-Aldrich, Hamburg, Germany) served as negative and positive controls, respectively. Spots of IFN-γ-positive cells were counted and analyzed and the results were expressed as the number of spots per well (spw). The mean number of spots in the negative control was subtracted from the mean number of spots in the antigen wells. The cut-off value for a positive response was ≥5 spw in healthy donors and ≥2 spw in HAdV-infected patients.

### T-cell proliferation assay

The proliferative capacities of HAdV-specific T cells induced by the various peptide candidates (Table 1, highlighted in italic) were analyzed by carboxyfluorescein succinimidyl ester (CFSE) dilution assay (Invitrogen, Darmstadt, Germany). 5 × 10^5^ CFSE-labeled PBMCs were stimulated with 10 µg/ml peptide for 7 days. Unstimulated T cells were used as negative controls. Spontaneous T-cell proliferation in unstimulated controls was subtracted from the specific values in peptide-stimulated cultures. Cells were stained with allophycocyanin (APC)-conjugated anti-CD8 mAb and PE-Cy7-conjugated anti-CD3 mAb (all BD Biosciences). The distribution of viable and dead cells was analyzed by 7-amino-actinomycin D (7-AAD) staining (BD Biosciences). At least 50,000 events were acquired in the 7-AAD^−^ gate. CFSE^+/−^CD3^+^ and CFSE^+/−^CD8^+^ T-cell populations were gated based on the scatter properties of viable 7-AAD^−^CD3^+^ or 7-AAD^−^CD3^+^CD8^+^ T lymphocytes.

### Immunophenotyping and detection of HAdV-specific T cells

A comprehensive analysis of the phenotype and specificity of HAdV-specific T cells was performed with freshly isolated PBMCs and in vitro expanded HAdV-specific T cells. PBMCs from healthy donors were stimulated for 7 days with 10 µg/ml peptide (Table 1, highlighted in italic), and restimulated for another 7 days with irradiated autologous peptide-loaded PBMCs. Flow cytometric analysis of the CD8^+^ T-cell phenotype was performed using cells stained with the mAbs anti-CD3 peridinin chlorophyll protein (PerCP), anti-CD8 APC (BD Biosciences), anti-CD19 FITC, anti-CD62L APC-Cy7, and anti-CD45RA PE-Cy7 (BioLegend) to assess the frequencies of naïve T cells (T_N_; CD62L^+^ CD45RA^+^), central memory T cells (T_CM_; CD62L^+^ CD45RA^−^), effector memory T cells (T_EM_; CD62L^−^ CD45RA^−^), and terminally differentiated effector memory T cells (T_EMRA_; CD62L^−^ CD45RA^+^).

In addition, specificities and frequencies of CD8^+^ T cells against the peptide candidates A01Penton_STDV_, A02Hexon_TLLY_, and the positive control A01Hexon_TDLG_ (Table 1) were detected by pMHC multimer staining using R-PE-conjugated multimeric Pro5 pentamers (ProImmune). At least 100,000 events were acquired in the lymphocyte gate, which was set based on the light scatter properties scatter properties of lymphocytes and on CD3^+^ T-cell populations. To be considered positive, the sample had to (1) be a well-defined cell population and/or (2) contain ≥0.3 % pentamer^+^CD8^+^ T cells.

### Cytotoxic activity of peptide-induced HAdV-specific T cells

The cytotoxicity of in vitro expanded peptide-specific T cells (day 14) was assessed in a non-radioactive flow cytometric assay using autologous CFSE-labeled peptide-loaded PBMCs as target cells. In order to exclude alloreactivity, unloaded CFSE-labeled PBMCs were further used as target cells, in which the basal cytotoxic activity of effector T cells against the unloaded target cells was subtracted from the specific cytotoxic values. Briefly, effector T cells were incubated with target cells at effector to target (E:T) ratios of 10:1, 30:1 and 60:1. Target cell lysis was assessed after 5 h using 7-AAD staining.

In addition, antiviral T-cell degranulation was determined as a surrogate marker of cytotoxicity by testing for CD107a cell surface expression by flow cytometry. In vitro expanded HAdV-specific T cells were incubated with 10 µg/ml peptide and PE-Cy7-conjugated anti-CD107a mAb (BioLegend) at 37 °C and 5 % CO_2_. After 1 h of incubation, monensin (1:1000, BioLegend) was added and cells were incubated for further 4 h before staining with anti-CD3 PerCP and anti-CD8 APC.

### Enrichment of HAdV peptide-specific T cells

The enrichment efficiency of in vitro expanded HAdV peptide-specific T cells (day 14) was assessed using the cytokine secretion assay (Miltenyi Biotec). Aliquots of cell fractions before enrichment (“Origin”) and after enrichment (“Eluate”) were used for detailed analysis of IFN-γ-secreting viable HAdV-specific T-cell subsets by multicolor flow cytometry. In addition to anti-IFN-γ PE (Miltenyi Biotec), cells were stained with anti-CD45 APC-Cy7, anti-CD56 PE-Cy7, anti-CD3 FITC, anti-CD8-APC mAbs, and 7-AAD (all BD Biosciences). At least 10,000 events were acquired in the viable CD45^+^7AAD^−^ leukocyte gate.

### Statistical analysis

Statistical analysis was performed using the Prism v5.02 software (GraphPad, San Diego, California, USA). The results are displayed as mean ± standard deviation (SD). Generated data were analyzed using non-parametric Mann–Whitney U test. Significance levels were calculated and expressed as p values (*p < 0.05, **p < 0.01, ***p < 0.001).

## Results

### Selection of potential HLA-restricted peptide epitopes within hexon and penton protein

A total of 947 hexon- and penton-derived epitope candidates restricted to HLA-types A*01, A*02, A*03, and B*08 were identified by the computer algorithms SYFPEITHI, BIMAS, and NetChop (Fig. 1). Nineteen (2 %) of the highest scoring sequences (six HLA-A*01-, five HLA-A*02-, two HLA-A*03-, and six HLA-B*08-resticted peptides; Table 2, [13, 20–23]) were synthesized. All three algorithms yielded comparably high prediction scores for seven of the 19 peptide epitopes (A02Hexon_TLLY_, B08Hexon_GLRY_, A01Penton_STDV_, A01Penton_SSDI_, A02Penton_ILHT_, B08Penton_NTKY_, and B08Penton_DSKK_): the SYFPEITHI score was 23–35, the BIMAS rank 1–2, and NetChop rating “strong binder (SB)” (Table 2). In comparison to the known immunodominant peptide epitopes of HAdV (A01Hexon_TDLG_) and of cytomegalovirus (A01pp65_YSEH_, and A02pp65_NLVP_), we detected (Additional file 1: Figure S1A) the highest binding affinity for A01Penton_STDV_ (FI 0.13), A01Penton_SSDI_ (FI 0.11), A02Penton_ILHT_ (FI 1.29) and A02Penton_ALGI_ (FI 1.29) by in vitro T2 peptide binding assay. The peptide binding results generated by computer and in vitro analyses were comparable. Because immunodominant peptides bound to HLA molecules were shown to be more stable than non-immunogenic peptides [33, 37], we further investigated the peptide-HLA class I stability of peptides of the database-available HLA types A*01, A*02, and A*03 using the CBS NetMHC_stab_ algorithm (Table 2). HLA class I peptide complexes with a predicted half-life (t_1/2_) > 2 h are defined as very stable [33]. This criterion was met by 7/14 epitopes (50 %) restricted to HLA-A*01, -A*02, and -A*03, including two highly stable (HS, t_1/2_ ≥ 6 h) and five weakly stable (WS, t_1/2_ ≥ 2 h) epitopes.

**Table 2:** Prediction results for the investigated HAdV epitope candidates

Abbreviation	Prediction score				
	SYFPEITHI [score]	BIMAS [rank]	NetChop		
			NetMHC_cons_ [BL]^b^	NetMHC [BL]^b^	NetMHC_stab_ [SL]^c^, T_1/2_^a^
A01Hexon_TDLG_	20	0.125 [134]	11052.58	13301	0.63
A01Hexon_TNDQ_	28	6.25 [11]	677.84 [SB]	1182	2.08 [WS]
A01Hexon_QNDP_	27	125 [1]	1691.19 [WB]	3899	2.97 [WS]
*A02Hexon*_*TLLY*_	27	3432.948 [1]	3.25 [SB]	3 [SB]	14.1 [HS]
A02Hexon_TLAV_	25	69.552 [15]	649.13	513	1.09
B08Hexon_GLRY_	32	160 [1]	42.02 [SB]	40 [SB]	–
*B08Hexon*_*DLQD*_	26	24 [2]	2339.71	3791	–
*A01Penton*_*STDV*_	35	312.5 [1]	5.9 [SB]	7 [SB]	4.86 [WS]
A01Penton_SNDS_	28	6.25 [8]	953.12 [WB]	2784	1.74
A01Penton_SSDI_	33	187.5 [1]	8.07 [SB]	9 [SB]	4.23 [WS]
A01Penton_LTDH_	21	6.25 [9]	43.65 [SB]	30 [SB]	1
*A02Penton*_*ILHT*_	23	271.948 [2]	16.75 [SB]	18 [SB]	5.72 [WS]
A02Penton_ALGI_	23	69.552 [5]	69.13 [WB]	64 [WB]	7.77 [HS]
A02Penton_GNIP_	20	>20	16673.41	17051	0.5
A03Penton_VLES_	25	9 [4]	1073.58	1099	0.7
A03Penton_LLPG_	23	3 [5]	27426.93	21324	0.22
B08Penton_NTKY_	29	80 [2]	39.6 [SB]	26 [SB]	–
*B08Penton*_*DSKG*_	28	160 [1]	755.3 [WB]	799	–
B08Penton_LTKD_	32	120 [2]	2134.13	4050	–
*B08Penton*_*DSKK*_	28	160 [1]	130.88 [SB]	80 [WB]	–

Prediction results for 19 epitope candidates (plus one reference epitope, A01Hexon_TDLG_) predicted for frequent HLA class I alleles and clinically relevant HAdV types using three different prediction tools (SYFPETHI, BIMAS, and NetChop). Epitope candidates were selected with respect to their predicted HLA binding affinity (*BL* binding level) and peptide-HLA complex stability (*SL* stability level). Epitopes were classified as weak binders (*WB threshold* 500 nM) or strong binders (*SB threshold* 50 nM) with either weak stability (*WS threshold* 2 h) or high stability (*HS threshold* 6 h) according to the predicted scores.

Pre-classified immunodominant peptide candidates are highlighted in italic

^a^Estimated half-time (t_1/2_) of dissociation (in hours)

^b^Estimated peptide binding affinity to HLA alleles as IC_50_ value (in nM)

^c^Estimated stability of peptide-MHC I complexes as IC_50_ value (in nM), and IC_50_ = half-maximum inhibitory concentration

### Identification of specific IFN-γ responses to the HAdV peptide candidates

**Fig. 2:**
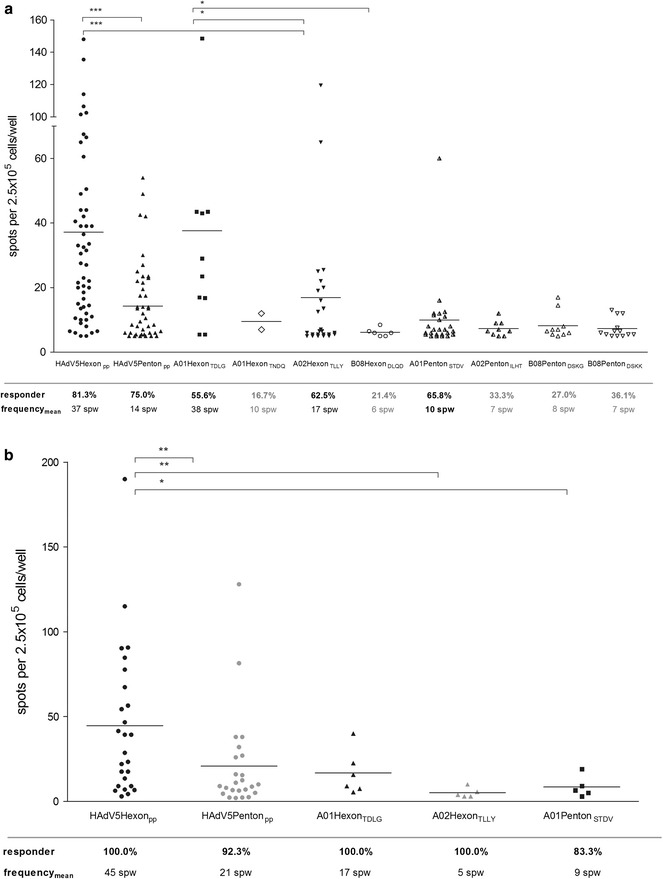
Screening of HAdV-specific T-cell responses by IFN-γ EliSpot. **a** IFN-γ EliSpot was performed in 64 healthy donors to evaluate specific T-cell responses to overlapping HAdV hexon (HAdV5Hexon_pp_) and penton peptide pools (HAdV5Penton_pp_), four hexon-derived peptides (A01Hexon_TDLG_, A01Hexon_TNDQ_, A02Hexon_TLLY_, B08Hexon_DLQD_), and four penton-derived peptides (A01Penton_STDV_, A02Penton_ILHT_, B08Pentn_DSKG_, B08Penton_DSKK_). **b** IFN-γ EliSpot analysis of 26 HAdV-infected HSCT recipients for specific T-cell responses to overlapping HAdV hexon (HAdV5Hexon_pp_) and penton peptide pools (HAdV5Penton_pp_) and to the peptides A01Hexon_TDLG_, A02Hexon_TLLY_ and A01Penton_STDV_. T-cell responses were assessed in HAdV-infected post-transplant patients. The highest T-cell response is shown for each tested patient. PBMCs cultured with medium alone or in the presence of 1 µg/ml staphylococcal enterotoxin B (SEB) served as negative and positive controls, respectively. IFN-γ EliSpot results are expressed as the number of IFN-γ spots per well (spw). The cut-off value for a positive response was ≥ 5 spw in healthy donors and ≥2 spw in HAdV-infected HCST recipients. Only data and mean frequencies from positive responder are shown. *Asterisks* indicate statistically significant differences between the antigens (*p < 0.05, **p < 0.01, ***p < 0.001)

Our analysis of specific memory T-cell responses to the control antigens HAdV5Hexon_pp_, HAdV5Penton_pp_, and A01Hexon_TDLG_ revealed high response rates in healthy donors (Fig. 2a) and HAdV-infected HSCT recipients (Fig. 2b). A total of 19 peptide candidates were selected and analyzed by IFN-γ EliSpot for their capacity to induce HAdV-specific T-cell responses. Seven elicited a specific T-cell response, defined by a cut-off of ≥5 spw (Table 1). The response rates ranged from 16.7 to 65.8 % (Fig. 2a). Pre-classification of peptide candidates into non-immunodominant or immunodominant was performed according to the number of responders (≥20 %).

The A01Hexon_TNDQ_ was pre-classified as non-immunodominant (16.7 % responder), whereas peptides B08Hexon_DLQD_, A02Penton_ILHT_, B08penton_DSKG_, B08Penton_DSKK_ (n = 4, Table 1) were pre-classified as low immunodominant. T cells from ≥50 % of healthy donors recognized the peptides A02Hexon_TLLY_ and A02Penton_STDV_ and were therefore pre-classified as highly immunodominant. The mean number of IFN-γ^+^ spots ranged from 6 to 17 spw, and was highest for A02Hexon_TLLY_ (17 spw) and A01Penton_STDV_ (mean 10 spw).

The response to the hexon control antigens (HAdV5Hexon_pp_: 81.3 % responder, 37 spw; A01Hexon_TDLG_: 55.6 % responder, 38 spw) was comparably high in healthy donors. For HAdV5Penton_pp_ we found a positive T-cell response in 75 % of donors (14 spw) and T-cell frequencies which were not significantly higher compared to the new A01Penton_STDV_ epitope.

In HAdV-infected HSCT patients, the immunogenicity of both peptide epitopes A02Hexon_TLLY_ and A01Penton_STDV_ was further assessed to confirm their clinical relevance (Fig. 2b). Specific T-cell responses against the control antigens HAdV5Hexon_pp_ (45 spw) and A01Hexon_TDLG_ (17 spw) were found in 100 % of patients. All HLA-A*02-positive patients (5/5) developed a HAdV-specific T-cell response against A02Hexon_TLLY_ (≥2 spw, 5 spw). For A01Penton_STDV_ we found a positive T-cell response in 5/6 (83.3 %) of patients (9 spw), while 24/26 (92.3 %) of all patients developed a HAdV5Penton_pp_-specific T-cell response (21 spw).

Comparing both overlapping peptide pools, T-cell responses to hexon were significantly higher (healthy donors: 2.6-fold, patients: 2.1-fold) than those to penton. Furthermore, T cells from most donors and patients recognized HAdV5Hexon_pp_ and A01Hexon_TDLG_ at higher frequencies than A02Hexon_TLLY_, but showed no significant frequency differences between HAdV5Penton_pp_ and the peptide candidate A01Penton_STDV_.

### Validation of pre-classified immunodominant adenoviral CTL epitopes

To find the optimal concentrations of the six pre-classified immunodominant peptides (Table 1, highlighted in italic) for T-cell immunoassays, we determined the sensitizing peptide concentration required to elicit 50 % of maximal T-cell responses (SD_50_) using the IFN-γ EliSpot assay (Fig. 1; Additional file 1: Figure S1B). The SD_50_ ranged from 5 to 10 µg/ml for B08Hexon_DLQD_ (n = 2) and B08Penton_DSKG_ (n = 2), and from 10 µg/ml to 50 µg/ml for A02Hexon_TLLY_ (n = 3), A01Penton_STDV_ (n = 2), A02Penton_ILHT_ (n = 2), and B08Penton_DSKK_ (n = 2). Therefore, T-cell immunoassays were performed at a final peptide concentration of 10 µg/ml.

#### Highly proliferative capacity of peptide-induced HAdV-specific T cells

**Fig. 3:**
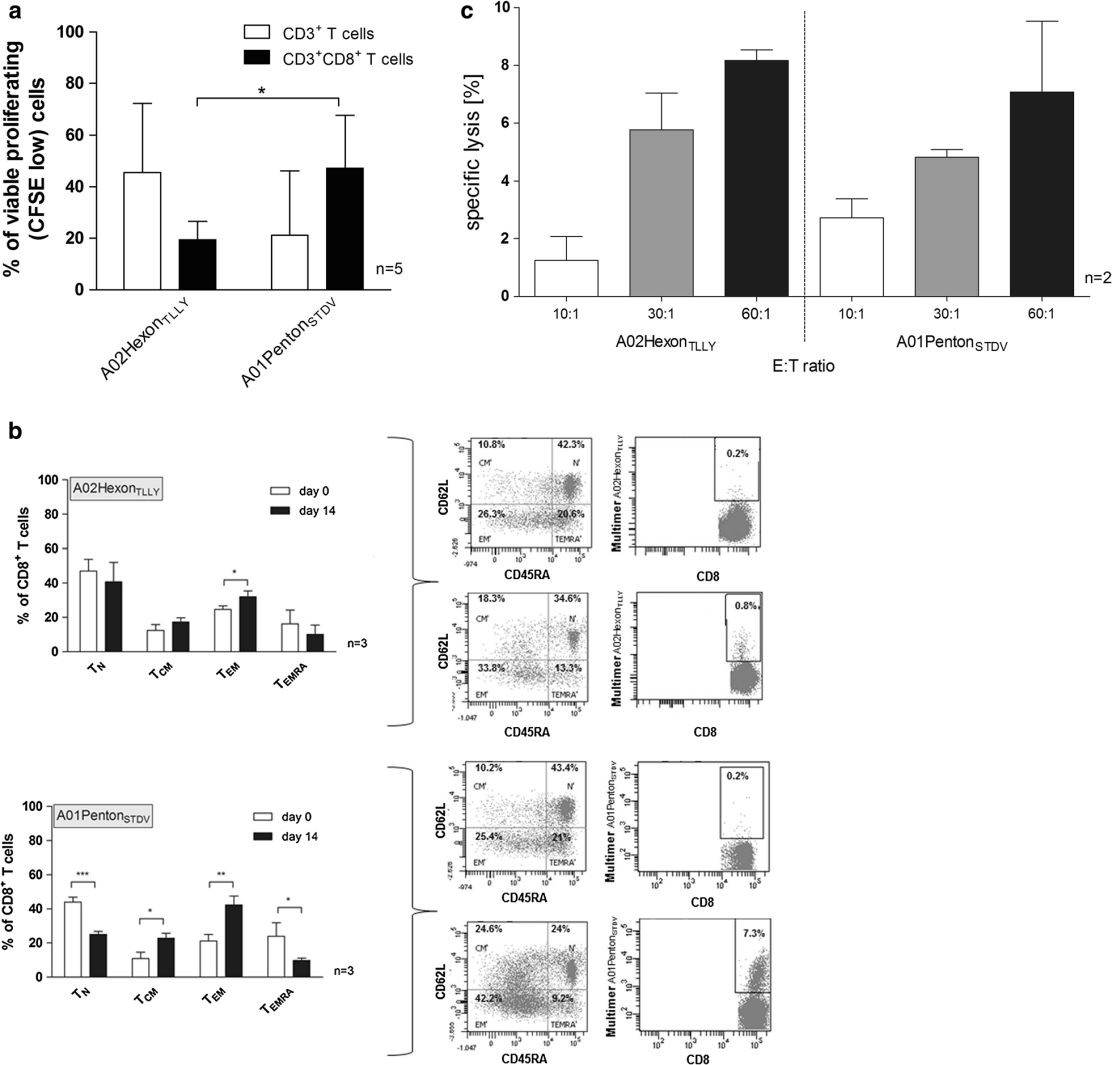
Analysis of HAdV-specific T-cell responses to the immunodominant epitopes A02Hexon_TLLY_ and A01Penton_STDV_ in healthy donors. HAdV-specific T cells induced by A02Hexon_TLLY_ and A01Penton_STDV_ were characterized in relation to **a** proliferative capacity of peptide-specific T cells within CD3^+^ and CD3^+^CD8^+^ T-cell populations **b** phenotype and frequency of HAdV-specific CD8^+^ T cells, (C) cytotoxicity of CD8^+^ T cells by multicolor flow cytometry. **a** PBMCs from healthy donors were labeled with carboxyfluorescein succinimidyl ester (CFSE) and stimulated over 7 days with A02Hexon_TLLY_ or A01Penton_STDV_, respectively and analyzed for the frequency of viable proliferative CD3^+^ and CD3^+^CD8^+^ T cells (CFSE low). The proliferative capacity of unstimulated CSFE-labeled PBMCs (negative control) was subtracted from the values for peptide-stimulated PBMCs. Results of independent experiments (n = 5) are expressed as mean ± SD. **b** Frequency of A02Hexon_TLLY_- and A01Penton_STDV_-specific CD8^+^ T cells and their four subsets—naïve (T_N_), central (T_CM_), effector memory (T_EM_), and terminally differentiated effector memory (T_EMRA_) T cells—from healthy donors before (day 0) and after two restimulation cycles (day 14), including *dot plots* for phenotype and multimer^+^CD8^+^ T cells of one representative donor (*upper right*). Results of independent experiments (n = 3) are expressed as mean ± SD. **c** Cytotoxic activity of expanded A02Hexon_TLLY_- and A01Penton_STDV_-specific T cells from healthy donors (day 14, effector cells, *E*) was analyzed in five-hour cytotoxicity assays using CFSE-labeled peptide-loaded/unloaded PBMCs as target cells (*T*). Effector T cells were co-cultured with target cells at an E:T ratio of 10:1, 30:10, or 60:1, respectively. Basal cytotoxic activity of effector T cells induced by A02Hexon_TLLY_ or A01Penton_STDV_ against the unloaded target cells was subtracted from the cytotoxic T-cell values against peptide-loaded PBMCs. Results (n = 2) are expressed as the mean percentage of target cell lysis ± SD. *Asterisks* shown in the figure indicate statistically significant differences between T-cell proliferation, phenotype and cytotoxicity in response to A02Hexon_TLLY_- and A01Penton_STDV_ (*p < 0.05, **p < 0.01, ***p < 0.001, SD, standard deviation)

The proliferation profile of HAdV-specific T cells in response to A02Hexon_TLLY_ and A01Penton_STDV_ (n = 5, Fig. 3a) was assessed 7 days after in vitro stimulation. A mean frequency of 33.3 % CFSE^low^CD3^+^ (A02Hexon_TLLY_: mean 45.5 %, A01Penton_STDV_: mean 21.1 %) and CFSE^low^CD3^+^CD8^+^ T cells (A02Hexon_TLLY_: mean 19.4 %, A01Penton_STDV_: mean 47.2 %) was detected, while A01Penton_STDV_-stimulation resulted in 2.4-fold higher CD3^+^CD8^+^ T-cells proliferation (p < 0.05) than A02Hexon_TLLY_. The other peptide candidates also resulted in high T-cell proliferation capacities (Table 1; Additional file 2: Figure S2): B08Hexon_DLQD_ induced the highest proliferative capacity of CD3^+^ T cells (mean 52.9 %), while B08Penton_DSKK_ induced the highest CD3^+^CD8^+^ T-cell proliferation (mean 39.1 %).

#### Differentiation phenotype of HAdV peptide-specific CD8^+^ T cells

After in vitro stimulation, A02Hexon_TLLY_- and A01Penton_STDV_-specific CD8^+^ T cells displayed a phenotype characterized by a loss of CD45RA (Fig. 3b). Phenotypic analysis revealed that the proportion of CD8^+^ T-cell subsets in response to stimulation with A02Hexon_TLLY_ and A01Penton_STDV_ significantly changed during T-cell expansion. The highest phenotypic changes were observed in CD8^+^ T-cell responses to A01Penton_STDV_, with a significant decrease in T_N_ (mean 44.1 % to 25.1 %, p < 0.001) and T_EMRA_ (mean 23.9 % to 9.8 %, p < 0.05), and significantly increased frequencies of T_CM_ (mean 10.8 % to 22.9 %, p < 0.05) and T_EM_ (mean 21.2 % to 42.3 %, p < 0.01 %). Stimulation with A02Hexon_TLLY_ also resulted in a significant increase in CD8^+^ T_EM_ (mean 24.6 % to 32 %, p < 0.05 %). Higher frequencies of cytotoxic A02Hexon_TLLY_-specific (mean 5-fold) and A01Penton_STDV_-specific CD8^+^ T cells (mean 11-fold) were further detected after T-cell expansion using the respective pentamers (Fig. 3b). Expansion of HAdV-specific CD8^+^ T cells in response to the other peptide candidates (Additional file 2: Figure S2B) resulted in a comparable, differentiated phenotype with decreased frequencies of T_N_ (mean 1.2-fold) and T_EMRA_ (mean 1.7-fold) and increased frequencies of T_CM_ (mean 1.5-fold) and T_EM_ (mean 1.3-fold).

#### Cytotoxic capacity of expanded HAdV peptide-specific CD8^+^ T cells

The cytotoxic activity of expanded HAdV-specific CTLs against the six pre-classified immunodominant peptide candidates (Table 1, highlighted in italic) was analyzed to evaluate their functionality and specificity. Comparison of peptide candidates A02Hexon_TLLY_ and A01Penton_STDV_ (Fig. 3c) showed that the highest cytotoxicity occurred at an E:T ratio of 60:1 for A02Hexon_TLLY_-specific CTLs (8.2 ± 0.4 %). The cytotoxic capacities in response to the other candidates (Additional file 2: Figure S2C) were similarly high, while the highest percentage of specific target lysis was detected for B08Hexon_DLQD_-specific CTLs at an E:T ratio of 60:1 (10.6 ± 8.5 %).

Increased expression of IFN-γ (cytotoxic marker) and CD107a (degranulation marker) further verified the peptide-specific function and specificity of activated CD8^+^ CTLs. A02Hexon_TLLY_ and A01Penton_STDV_ generated the highest IFN-γ^+^ T-cell responses (Fig. 2a), while B08Penton_DSKG_ (18.7 %) and A01Penton_STDV_ (15.8 %) induced the highest frequencies of CD107a-positive CD3^+^CD8^+^ T cells (Additional file 2: Figure S2D). Compared to unstimulated CD3^+^CD8^+^ T cells, the highest-fold increases in CD107a-positive CD3^+^CD8^+^ T cells was observed in response to the peptides A02Hexon_TLLY_ (5.7-fold increase) and B08Hexon_DLQG_ (3.3-fold increase).

#### A01Penton_STDV_ and A02Hexon_TLLY_ induce the expansion of HAdV-specific CD8^+^ CTLs

**Fig. 4:**
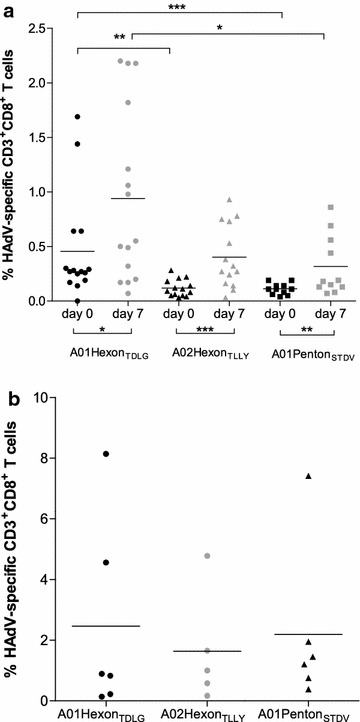
Frequencies of HAdV-specific T cells in healthy donors and HAdV-infected HSCT recipients. Percentage of HAdV-specific CD3^+^CD8^+^ T cells as detected by pentamer staining. **a** PBMCs from healthy donors were analyzed for A01Hexon_TDLG_-, A02Hexon_TLLY_- and A01Penton_STDV_-specific CD3^+^CD8^+^ T cells before (day 0) and after stimulation with the peptides A01Hexon_TDLG_, A02Hexon_TLLY_ or A01Penton_STDV_, respectively (day 7). Frequencies of A01Hexon_TDLG_-, A02Hexon_TLLY_- and A01Penton_STDV_-specific CD8^+^ T cells, as detected by pentamer staining, are expressed as mean frequencies of HAdV-specific CD3^+^CD8^+^ T cells. **b** T-cell responses were assessed in HAdV-infected patients. Highest frequencies of A01Hexon_TDLG_, A02Hexon_TLLY_-, and A01Penton_STDV_-specific CD8^+^ T cells as detected by pentamer staining, were shown for each patient and are expressed as mean frequencies of HAdV-specific CD3^+^CD8^+^ T cells. CD8^+^ T cells were stained with PE-labeled pentamers. The results of independent experiments are expressed as mean HAdV-specific CD3^+^CD8^+^ T-cell frequencies. *Asterisks* indicate statistically significant differences between the HAdV peptides (*p < 0.05, **p < 0.01, ***p < 0.001)

Healthy donors were tested for precursor frequencies of circulating A02Hexon_TLLY_- and A01Penton_STDV_-specific CD8^+^ T cells and in vitro T-cell expansion efficiency (Fig. 4a). The frequencies of A02Hexon_TLLY_-(0.12 ± 0.08 %, p < 0.01) and A01Penton_STDV_-(0.11 ± 0.05 %, p < 0.001) specific CD8^+^ T-cell precursors in freshly isolated PBMCs were significantly lower than the frequencies of circulating A01Hexon_TDLG_-specific CD8^+^ T cells (0.46 ± 0.48 %, positive controls); however, A01Penton_STDV_ and A01Hexon_TDLG_ resulted in significant differences after in vitro expansion (p < 0.05). The numbers of responders in freshly isolated PBMCs (A01Hexon_TDLG_: 66.7 %, A02Hexon_TLLY_: 64.3 %, A01Penton_STDV_: 63.6 %) were comparable to those detected by IFN-γ EliSpot. After in vitro expansion, significantly higher frequencies of HAdV-specific CD8^+^ T cells were detected in response to all investigated HAdV peptides; rates ranged from 0.32 to 0.94 %, representing a 2.1 to 3.4-fold increase as compared to freshly isolated PBMCs.

Furthermore, the frequencies of antigen-specific CD8^+^ T cells against A02Hexon_TLLY_ and A01Penton_STDV_ in peripheral blood from HAdV-infected patients were monitored to validate their clinical relevance (Fig. 4b). Results obtained by pentamer staining correlated with those from the EliSpot assay. Increased frequencies of HAdV-specific CD8^+^ T cells against A01Hexon_TDLG_ (mean 2.46 %, n = 6), A02Hexon_TLLY_ (mean 1.64 %, n = 5) and A01Penton_STDV_ (mean 2.19 %, n = 6) were detected with no significant differences between the three peptide epitopes. The generated pentamer results emphasize the differentiated CD45RA^neg^ phenotype of expanded peptide-specific CTLs.

### High enrichment efficiency of A02Hexon_TLLY_- and A01Penton_STDV_-specific T cells

**Fig. 5:**
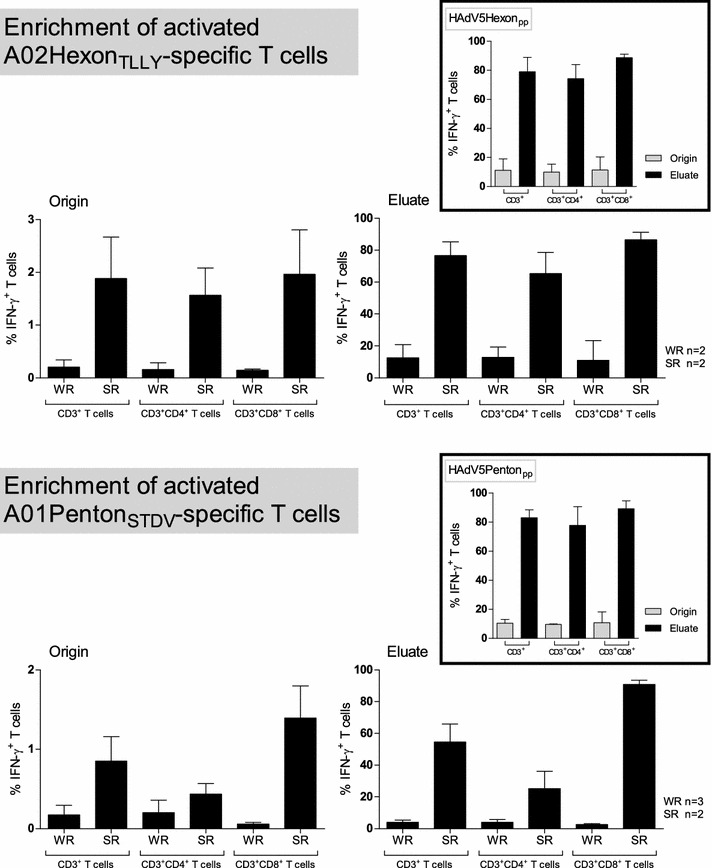
Enrichment of functional A01Penton_STDV_- and A02Hexon_TLLY_-specific T cells by IFN-γ-based cytokine secretion assay. HAdV-specific T cells were induced by stimulating PBMCs from healthy donors with A02Hexon_TLLY_, A01Penton_STDV_, HAdV5Hexon_pp_ or HAdV5Penton_pp_, respectively. Stimulated T cells were isolated using IFN-γ-based cytokine secretion assay. The frequency of IFN-γ-secreting cells among CD3^+^, CD3^+^CD4^+^ and CD3^+^CD8^+^ T cells in the CSA fractions before (“Origin”) and after (“Eluate”) enrichment were determined by multicolor flow cytometry. The results of the peptide-specific T-cells enrichment process for weak responders (WR, <0.3 % IFN-γ^+^CD3^+^ T cells) and strong responders (SR, >0.3 % IFN-γ^+^CD3^+^ T cells) are shown as mean ± standard deviation (SD)

The function of in vitro expanded HAdV-specific T cells was evaluated by CSA in order to determine how quickly and strongly HAdV-specific T cells secreted IFN-γ in response to A02Hexon_TLLY_ and A01Penton_STDV_, respectively. IFN-γ^+^ T-cell populations in healthy donors were investigated by multicolor flow cytometry before and after enrichment (Fig. 5; Additional file 3: Figure S3). According to the peptide-specific CD3^+^ T-cell frequency in the CSA fraction before enrichment (“Origin”), donors were classified as either weak responders (WR < 0.3 % IFN-γ^+^CD3^+^ T cells, A02Hexon_TLLY_: n = 2, A01Penton_STDV_: n = 3) or strong responders (SR, > 0.3 % IFN-γ^+^CD3^+^ T cells, n = 2). The “Origin” CSA fraction contained 0.21 ± 0.13 % (WR) and 1.89 ± 0.78 % (SR) IFN-γ^+^CD3^+^ T cells, which could be enriched with a mean purity of 12.6 ± 8.22 % in WR and 76.7 ± 8.47 % in SR, including 11.0 ± 12.3 % (WR) to 86.6 ± 4.59 % (SR) of IFN-γ secreting CD3^+^CD8^+^ T cells (Fig. 5). The mean frequency of A01Penton_STDV_-specific IFN-γ^+^CD3^+^ T cells before (0.17 ± 0.12 % WR and 0.85 ± 0.35 % SR) and after enrichment (3.97 ± 1.43 % WR and 54.58 ± 11.30 % SR) was 1.5-fold lower than for A02Hexon_TLLY_, whereas the frequency of IFN-γ^+^CD3^+^CD8^+^ T cells was comparable (2.60 ± 0.62 % WR and 90.78 ± 2.67 % SR) to A02Hexon_TLLY_. The low enrichment efficiency of donors classified as weak responders reflects the low precursor frequency of antigen-specific CTLs (A02Hexon_TLLY_: 0.19 %, A01Penton_STDV_: 0.30 %), as determined by pentamer staining, which may lead to a low binding affinity of IFN-γ-secreting T cells in the CSA. In this context, starting with an antigen-specific CD8^+^ T-cell frequency >0.3 % (A02Hexon_TLLY_: 0.82 %, A01Penton_STDV_: 5.37 %, SR) resulted in the enrichment of highly pure IFN-γ-secreting T cells.

## Discussion

Adoptive T-cell immunotherapy has become a promising treatment option for patients with adenoviral infections after transplantation since these cells were shown to play a pivotal role in viral control and clearance. Accurate monitoring of the viral load and antiviral T-cell immunity is of great importance to effectively treat emerging or overt HAdV infections in post-transplant patients. Moreover, analysis of the specific T-cell repertoire in potential T-cell donors is essential to identify the most suitable donors for adoptive transfer and may prove helpful for donor choice in patients with pre-transplant viral conditions.

In this study, we assessed the immunogenicity of 19 potential adenoviral CTL epitopes identified by reverse immunology. Various epitope prediction algorithms were employed to pre-screen for immunodominant CTL epitopes. We found that the prediction accuracy for CD8^+^ T-cell epitopes was improved by combining different algorithms for peptide binding affinity and pMHC complex stability. Because prediction results do not reflect one-to-one correlation with experimental data, we validated the immunogenicity of the predicted epitopes in vitro. IFN-γ EliSpot evaluation of peptide-specific T-cell responses in PBMCs from healthy donors resulted in the pre-classification of four CTL epitopes as low immunodominant (response ≥20 %) and two as high immunodominant (response ≥50 %) (Table 1, highlighted in italic). Immunodominance of these six candidates was verified by T-cell immunoassays and T-cell immunomonitoring in HAdV-infected HSCT patients, which delineated the significance of the identified CD8^+^ T-cell epitopes in vivo. Guenther et al. identified the naturally presented HAdV epitopes LTDLGQNLLY and VPATGRTLVL [19], for which we obtained high prediction scores. These results underline the high prediction accuracy of HLA-class I epitopes obtained by the allele-specific prediction algorithms utilized in the present study.

### Description of the first identified penton-derived immunodominant CD8^+^ T-cell epitopes

Here, we were focused on the identification of peptide epitopes from HAdV penton, a major capsid protein. The penton base interacts with the other major capsid proteins (hexon and fiber) and is known to be involved in adenovirus cell entry [38]. Despite the variability of clinically relevant HAdV types, the penton sequence is composed of highly conserved regions and short hypervariable loops, similar to hexon [39].

Identification of immunodominant T-cell epitopes from these conserved regions represents an attractive approach to induce antiviral T cells that are cross-reactive with several potentially lethal HAdV types. Previous studies have demonstrated sequential or concomitant co-infections with different HAdV types in immunocompromised patients, which seem to result in the generation of recombinant HAdV types [6, 40, 41]. In the present and a previous study we identified HAdV types 1, 2, and 31 as the predominant pathogens in most pediatric patients with severe HAdV disease [7]. In our present study, 10 of 26 post-transplant patients were infected with type 31 of HAdV species A, and three of them were co-infected with type 2 of HAdV species C. The peptide epitopes identified in this study are shared among HAdV types and may thus facilitate viral clearance even in patients suffering from co-infection with different strains.

For the first time we identified three low immunodominant peptide epitopes (A02Penton_ILHT_, B08Penton_DSKG_, B08Penton_DSKK_) and one high immunodominant peptide epitope (A01Penton_STDV_) of the major HAdV capsid protein penton. Sequences of all identified immunodominant epitopes were highly conserved among the clinically relevant HAdV species C, with the exception of A31-derived B08Penton_DSKG_ (DSKGRSYNL), which only differs in one amino acid from the peptide B08Penton_DSKK_ (DSKKRSYNL) but is composed of the same amino acids at the conserved anchor positions P2 and P9. The sequence of the newly identified A01Penton_STDV_ is located between amino acid positions 76 and 84 of the penton protein of HAdV types 1, 2, and 5. Immune responses to the whole adenovirus, including the hexon protein, were dominated by memory CD4^+^ T cells. Interestingly, A01Penton_STDV_ was identified as a strong inducer of functional CD8^+^ CTLs (11-fold increase in A01Penton_STDV_-specific CD8^+^ CTLs), which could be efficiently enriched to a high frequency (90.4 % recovery), comparable to the HAdV5Penton_pp_ peptide pool (96.9 % recovery). In vitro expanded A01Penton_STDV_-specific CD8^+^ T cells showed a differentiated CD45RA^neg^ T-cell phenotype, with T_EM_ proved to be essential for effective clearance of and protection against HAdV infections, while T_CM_ demonstrated to be involved in preventive long-term immunity [13]. The immunogenicity of all four pre-classified immunodominant penton epitopes was verified by antiviral T-cell monitoring and in vitro T-cell immunoassay in healthy donors and patients.

### Evaluation of hexon-specific T-cell responses induced by the predicted CTL epitopes

In healthy donors, coordinate responses of hexon-specific CD4^+^ and CD8^+^ T cells were demonstrated to contribute to the control of HAdV infections, which persist as memory T cells [21]. Most hexon-derived epitopes are CD4 restricted [20, 22, 23]. We aimed to identify novel hexon-derived CD8^+^ T-cell epitopes by evaluating the immunogenic potential of six predicted CD8^+^ T-cell epitopes. Overall, we detected HAdV-specific T-cell responses to 3/6 predicted peptide candidates in healthy donors. B08Hexon_DLQD_ was thus pre-classified as a low immunodominant (≥20 % response) and A02Hexon_TLLY_ [13, 22, 23] as a high immunodominant CTL epitope (≥50 % response). The immunogenicity of both candidates was verified by T-cell monitoring and in vitro T-cell immunoassay. In accordance to already published data (Table 1), we found no or only minimal T-cell responses to the peptide candidates A01Hexon_TNDQ_ [21], A02Hexon_TLAV_ [13], and B08Hexon_GLRY_ [20]. In vitro expanded functional A02Hexon_TLLY_-specific CTLs showed a high proliferative and cytotoxic capacity and displayed a CD45RA^neg^ differentiated phenotype, which could be efficiently enriched to a significantly high frequency (85 % recovery), comparable to the HAdV5Hexon_pp_ peptide pool (95.5 % recovery).

Olive et al. demonstrated that additional three residues at each end of the immunodominant peptide Hexon_TLLY_ (15-mer peptide) resulted in a stronger CD4^+^ T-cell response. In addition, a lower peptide concentration for in vitro T-cell activation was required compared to the nonamer, indicating that the 15-mer peptide is an optimal CD4^+^ T-cell epitope [22, 23]. Guether et al. identified the decamer of the known immunodominant HLA-A01-restricted hexon-derived peptide (A01Hexon_TDLG_, LTDLGQNLLY) as a naturally presented T-cell epitope that induces peptide-specific T cells with a higher proliferative, cytotoxic, and IFN-γ-producing capacity than T cells specific for the shorter peptide TDLGQNLLY [19]. Therefore, we will evaluate the immunogenic potential of longer peptides from the novel identified low immunodominant B08Hexon_DLQD_ peptide in a future study.

### Comparison of HAdV-specific T-cell repertoires in healthy donors and HSCT recipients

Clinical relevance of the identified high immunodominant CTL epitopes A02Hexon_TLLY_ and A01Peton_STDV_ was further assessed in HAdV-infected HSCT recipients, compared to healthy donors and correlated to T-cell responses induced by HAdV5Hexon_pp_, HAdV5Penton_pp_, and the immunodominant A01Hexon_TDLG_. As expected, the majority of donors and patients had HAdV-specific T cells against hexon and penton, and the frequency of hexon-specific T cells was higher than that of penton-specific T cells in both cohorts. More than 50 % of all tested donors and patients had HAdV-specific T cells against A02Hexon_TLLY_, but at an up to 2.2 (donors) to ninefold (patients) lower frequency than the investigated control antigens HAdV5Hexon_pp_ and A01Hexon_TDLG_. For this reason, A02Hexon_TLLY_ can be classified as a high immunodominant CTL epitope but, as mentioned before, it has a lower immunogenic potential compared to the known HAdV5Hexon_pp_ and A01Hexon_TDLG_, as underlined by the higher immunogenicity of the 15-mer peptide DEPTLLYVLFEVFDV [22]. Interestingly, the immunogenic potential of A01Penton_STDV_, the novel penton-derived CTL epitope, was comparable to that of HAdV5Penton_pp_ in healthy donors and patients. This finding indicates that A01Penton_STDV_ is a major immunodominant penton-derived CTL epitope. In infected HSCT recipients we detected higher T-cell responses to the A01Penton_STDV_ than to the hexon-derived A02Hexon_TLLY_, which was the reverse in healthy donors. These data indicate that penton-specific T cells might be essential for viral control. Future studies including monitoring of T-cell functionality and specificity should be done to get more inside into the interplay between hexon- and penton-specific T cells in the first line of defense and/or long-term protective immunity against HAdV.

### Peptide-specific T-cell precursor frequencies and limits of detection

Low frequencies of circulating HAdV-specific T-cell precursors in peripheral blood make it difficult to detect HAdV-specific T cells, which can lead to false-negative results [36]. We were able to detect specific T-cell responses to the peptide epitopes A02Hexon_TLLY_ and A01Penton_STDV_ directly in freshly isolated PBMCs from healthy donors by IFN-γ EliSpot, albeit at quite low frequencies (A02Hexon_TLLY_: 17 spw, A01Penton_STDV_: 10 spw). Interestingly, the number of responders after peptide-restimulation was unchanged, indicating that no false-negative results were obtained in freshly isolated PBMCs compared to peptide-restimulated cells and that all tested donors were correctly identified as responders and non-responders. However, evaluation of A02Hexon_TLLY_ and A01Penton_STDV_ by pMHC multimer staining indicates the need for antigen-dependent T-cell expansion to avoid overlooking effector function of peptide-induced CTLs with very low precursor frequencies. Pentamer staining of freshly isolated PBMCs yielded false-negative results in 14.3 % A02Hexon_TLLY_-responders and 9.1 % of A02Penton_STD_-responders. Pentamer staining with in vitro stimulated cells resulted in more accurate identification of positive responders as a clearly defined peptide-specific CD8^+^ T-cell population with up to 3.3-fold higher frequencies. In this context, the highly sensitive IFN-γ EliSpot assay is a suitable technology for the detection of low precursor frequencies of circulating HAdV-specific T cells in peripheral blood. Conversely, for unambiguous identification of responders and non-responders by pentamer staining, we recommend short in vitro stimulation. The enrichment efficiency of antigen-specific T cells via CSA was further affected by the percentage of HAdV-specific T cells. The enrichment efficiency of A02Hexon_TLLY_- and A02Hexon_TLLY_-specific CD3^+^ T cells was lower (purity < 13 %) when the starting frequency was <0.3 %, whereas a high enrichment efficiency was obtained by using starting frequencies >0.3 % (<67 % purity). Therefore, short-term expansion protocols for the generation of higher numbers of functionally active HAdV-specific T cells, as described by Geyeregger et al., are a suitable option to generate sufficient numbers of HAdV-specific T cells for adoptive immunotherapy [12, 13]. In addition, manufacturing of clinical-grade HAdVPenton_pp_- and HAdVHexon_pp_-specific T cells in combination might be a promising option to provide sufficient numbers of effective HAdV-specific T cells for the adoptive T-cell transfer in immunocompromised patients.

## Conclusions

In summary, HAdV-specific T-cell responses to novel identified immunodominant CTL epitopes were observed in healthy donors, and the in vivo relevance of these identified CTL epitopes, as predicted by computer algorithms, was confirmed in HAdV-infected patients.

We describe the first immunodominant adenovirus CD8^+^ T-cell epitopes from the penton, of which A01Penton_STDV_ could be classified as the first high immunodominant CTL epitope discovered to date. HAdV-specific T-cell responses to A01Penton_STDV_ were comparable to those to the HAdV5Penton_pp_ overlapping peptide pool. The development of penton-specific T-cell immunity in HAdV-infected HSCT recipients suggested that, as an immunological target, the penton protein is not secondary to the hexon protein. Penton-specific T cells, particularly CD8^+^ CTLs, seem to be essential for effective defense against HAdV. Immunogenicity of the CTL epitope A02Hexon_TLLY_ was verified according to its prevalence in healthy donors and patients. A broad repertoire of immunodominant CD4^+^ and CD8^+^ T-cell epitopes appears to be crucial for improved immunomonitoring by means of precise quantitative assessment. This improvement will enhance the efficacy of adoptive immunotherapy by enabling the timely start of the adoptive transfer with the most suitable HAdV-specific T-cell populations.

## Additional files

10.1186/s12967-016-1042-2 Validation of peptide-binding affinity and concentration.

10.1186/s12967-016-1042-2 Analysis of HAdV-specific T-cell responses against the novel immunodominant T-cell epitope in healthy donors.

10.1186/s12967-016-1042-2 Enrichment of functional A02Hexon_TLLY_ and A01Penton_STDV_- specific T cells by IFN-γ-based cytokine secretion assay.
