# Supplementary material for: Discovery of immunodominant T-cell epitopes reveals penton protein as a second immunodominant target in human adenovirus infection
Source: J Transl Med. 2016 Oct 7;14:286. doi: 10.1186/s12967-016-1042-2 (PMC5055684; doi:10.1186/s12967-016-1042-2)
Supplement: Supplementary file 1 — 10.1186/s12967-016-1042-2 Validation of peptide-binding affinity and concentration. [file 12967_2016_1042_MOESM1_ESM.pdf]

Figure S1. Validation of peptide-binding affinity and concentration.

A

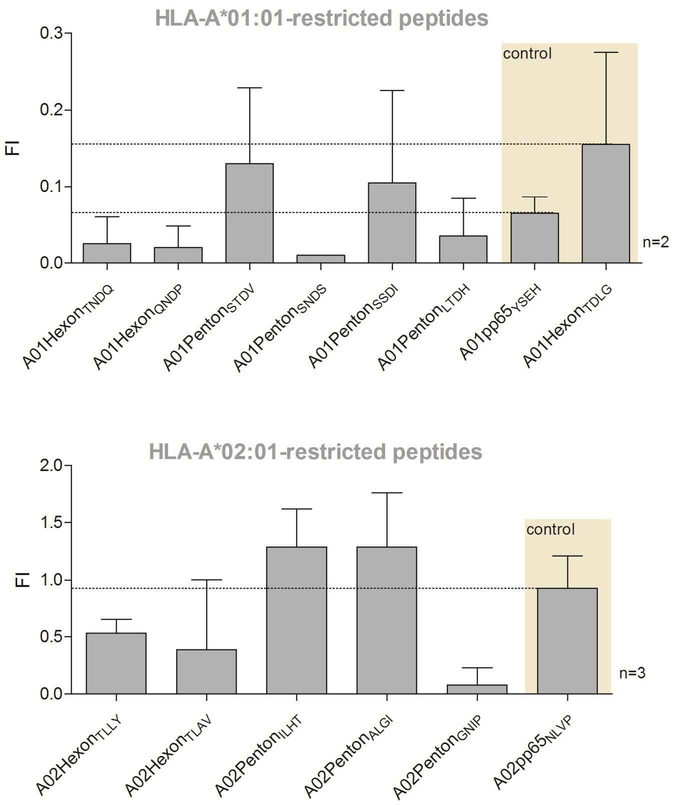

B

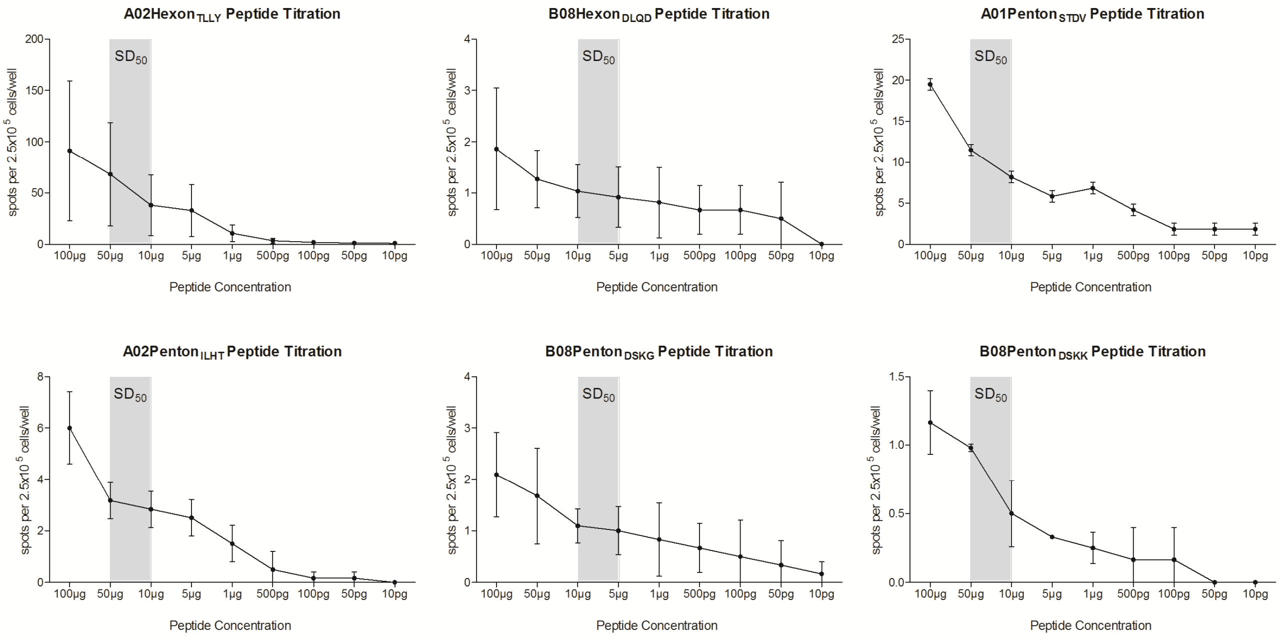

(A) The HLA binding affinity of the HAdV peptide candidates (nonamers, n=11) was analyzed by flow cytometry. Non-transduced (HLA-A*02^{+}) and HLA-A*01-transduced T2

cells were pulsed with the respective candidate peptide (50 μg/ml) plus human β2-microglobulin (5 μg/ml). T2 cells incubated without peptide served as controls. The immunogenic HLA-A*01- and A*02-restricted peptides (A01pp65$_{YSEH}$ and A02pp65$_{NLVP}$) from human cytomegalovirus (CMV) phosphoprotein 65 (pp65) as well as the immunogenic HLA-A*01-restricted hexon-derived peptide (A01Hexon$_{TDLG}$) served as positive controls. HLA-A*01 and HLA-A*02 expression levels on T2 cells were analyzed after 15-18 hours of incubation. The fluorescence index (FI) was calculated as the mean fluorescence intensity (MFI) of HLA-A*01:01 and HLA-A*02:01 on non-transduced and A01-transduced T2 cells, respectively. The results are expressed as the mean of n=2 (HLA-A*01) and n=3 (HLA-A*02) independent experiments ± standard deviation (SD). **(B)** For the definition of optimal T-cell stimulation conditions, concentration-dependent production of IFN-γ in response to the six identified immunodominant HAdV peptides (A02Heon$_{TLLY}$, B08Hexon$_{DLQD}$, B08Penton$_{DSKG}$, A01Penton$_{STDV}$, A02Penton$_{ILHT}$, and B08Penton$_{DSKK}$) was determined in peripheral blood mononuclear cells (PBMCs) from healthy donors by IFN-γ EliSpot as described previously [1, 2]. 2.5×10^5 PBMCs were plated in triplicate wells and incubated overnight with peptides titrated at final concentrations of 100 μg/ml to 10 pg/ml. PBMCs cultured in medium with or without 1 μg/ml staphylococcal enterotoxin B (SEB) served as positive and negative controls, respectively. IFN-γ EliSpot results are expressed as the number of IFN-γ spots per 2.5×10^5 cells/well (spw). Referring to the peptide concentration, the functional avidity of HAdV-specific CTLs was measured as the concentration of peptide required to elicit a half-maximal response (sensitizing dose, SD_{50}). The results of n=2 (B08Hexon$_{DLQD}$, B08Penton$_{DSKG}$, A01Penton$_{STDV}$, A02Penton$_{ILHT}$, and B08Penton$_{DSKK}$) and n=3 (A02Hexon$_{TLLY}$) independent experiments are expressed as the mean frequency of IFN-γ^+ T cells ± SD.

References

1. Sukdolak C, Tischer S, Dieks D, Figueiredo C, Goudeva L, Heuft HG, Verboom M, Immenschuh S, Heim A, Borchers S, et al: **CMV-, EBV- and ADV-specific T cell immunity: screening and monitoring of potential third-party donors to improve post-transplantation outcome.** *Biol Blood Marrow Transplant* 2013, **19:**1480-1492.
2. Tischer S, Dieks D, Sukdolak C, Bunse C, Figueiredo C, Immenschuh S, Borchers S, Stripecke R, Maecker-Kolhoff B, Blasczyk R, Eiz-Vesper B: **Evaluation of suitable target antigens and immunoassays for high-accuracy immune monitoring of cytomegalovirus and Epstein-Barr virus-specific T cells as targets of interest in immunotherapeutic approaches.** *J Immunol Methods* 2014, **408:**101-113.
