# Supplementary material for: Discovery of immunodominant T-cell epitopes reveals penton protein as a second immunodominant target in human adenovirus infection
Source: J Transl Med. 2016 Oct 7;14:286. doi: 10.1186/s12967-016-1042-2 (PMC5055684; doi:10.1186/s12967-016-1042-2)
Supplement: Supplementary file 2 — 10.1186/s12967-016-1042-2 Analysis of HAdV-specific T-cell responses against the novel immunodominant T-cell epitope in healthy donors. [file 12967_2016_1042_MOESM2_ESM.pdf]

Figure S2. Analysis of HAdV-specific T-cell responses against the novel immunodominant T-cell epitope in healthy donors.

A

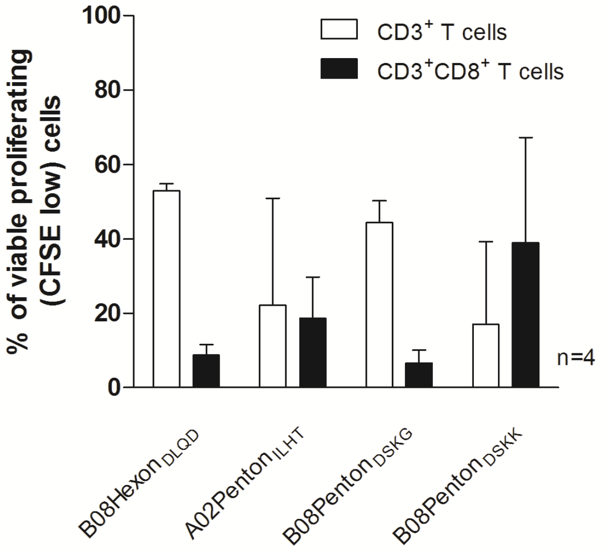

B

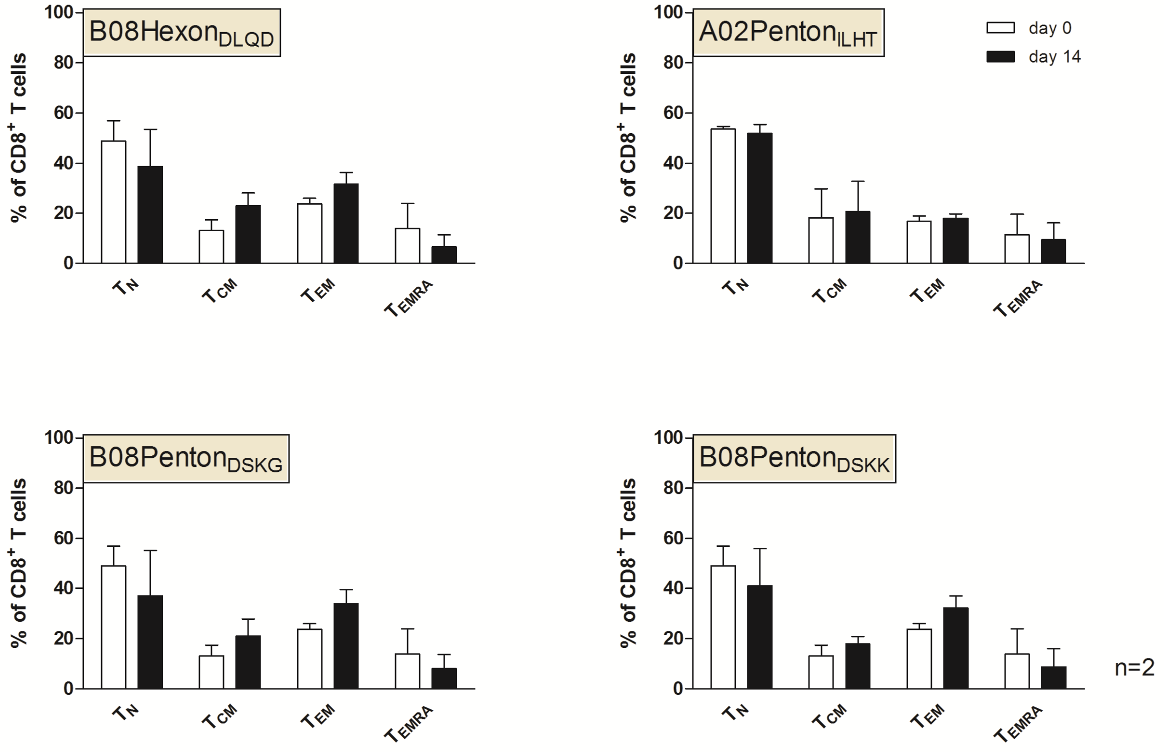

C

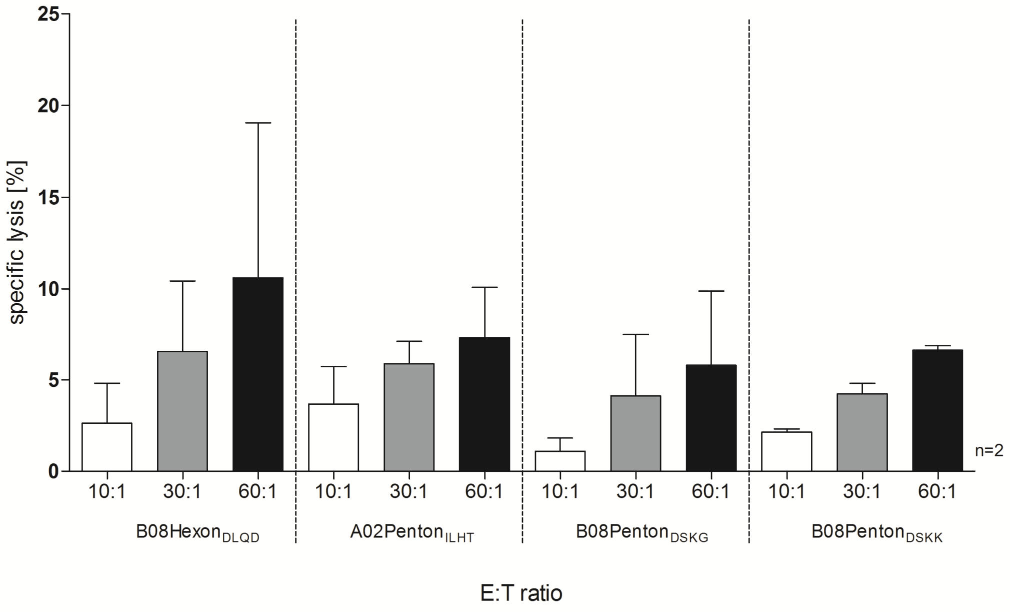

D

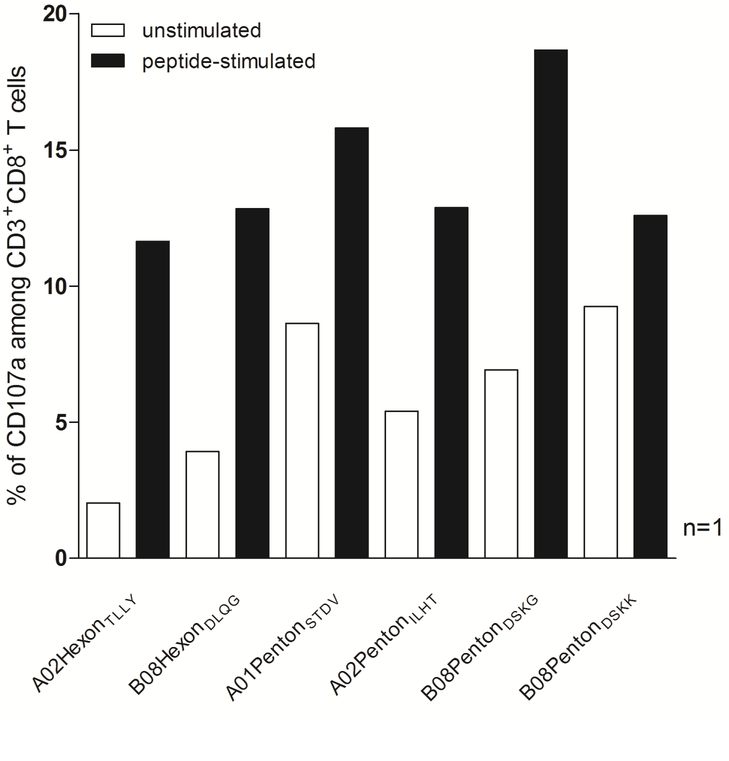

HAdV-specific T cells induced by the peptide epitopes identified as low immunodominant (A02Penton$_{ILHT}$, B08Hexon$_{DLQD}$, B08Penton$_{DSKG}$, and B08Penton$_{DSKK}$; Table 1A) were characterized by multicolor flow cytometry in relation to **(A)** proliferative capacity of peptide-specific T-cell populations, **(B)** CD8^{+} T-cell phenotype, and **(C)** cytotoxicity of CD3^{+}CD8^{+} T

cells. **(D)** Cytotoxic potential of $CD3^+CD8^+$ T cells in response to the investigated peptides (low-immunodominant: $A02Penton_{ILHT}$, $B08Hexon_{DLQD}$, $B08Penton_{DSKG}$, $B08Penton_{DSKK}$ and high-immunodominant: $A02Hexon_{TLLY}$, $A01Penton_{STDV}$) was assessed by using the CD107a degranulation assay. **(A)** Carboxyfluorescein succinimidyl ester (CFSE)-labeled PBMCs from healthy donors were stimulated for seven days with the investigated peptides and analyzed for the frequency of viable proliferative $CD3^+$ and $CD3^+CD8^+$ T cells (CFSE low). Unstimulated CFSE-labeled PBMCs served as negative controls. Results are expressed as mean ± SD (n=2). **(B)** Frequency of naïve (T_N), central memory (T_{CM}), effector memory (T_{EM}), and terminally differentiated effector memory (T_{EMRA}) $CD8^+$ T-cell subsets before (day 0) and after two restimulation cycles (day 14). Results are expressed as the mean of two independent experiments ± SD. **(C)** The cytotoxic activity of HAdV peptide-specific T cells (effector cells, E, day 14) was analyzed in five-hour cytotoxicity assays. Effector T cells were co-cultured with autologous PBMCs as target cells at E:T ratios of 10:1, 30:10, and 60:1, respectively. The basal cytotoxic activity of effector T cells induced by HAdV peptides against the unloaded target cells was subtracted from the cytotoxic T-cell values against peptide-loaded PBMCs. Results of two independent experiments are expressed as the mean percentage of target cell lysis ± SD. **(D)** The cytotoxic activity of HAdV-specific T cells induced by the identified immunodominant peptides (day 14) was verified by CD107a degranulation assay. CD107a expression data are shown for a representative donor (SD, standard deviation).
