# Supplementary material for: Discovery of immunodominant T-cell epitopes reveals penton protein as a second immunodominant target in human adenovirus infection
Source: J Transl Med. 2016 Oct 7;14:286. doi: 10.1186/s12967-016-1042-2 (PMC5055684; doi:10.1186/s12967-016-1042-2)
Supplement: Supplementary file 3 — 10.1186/s12967-016-1042-2 Enrichment of functional A02HexonTLLY and A01PentonSTDV- specific T cells by IFN-γ-based cytokine secretion assay. [file 12967_2016_1042_MOESM3_ESM.pdf]

Figure S3. Enrichment of functional A02Hexon$_{TLLY}$ and A01Penton$_{STDV}$- specific T cells by IFN-γ-based cytokine secretion assay.

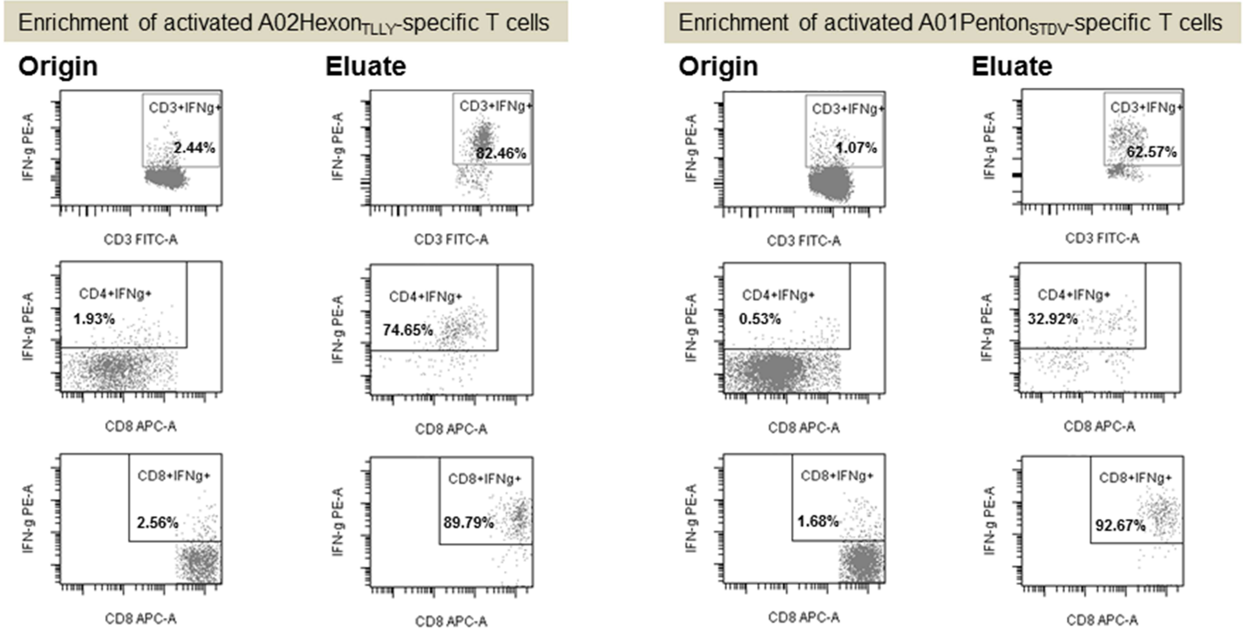

Functional peptide-specific T cells induced by stimulating PBMCs from healthy donors with the peptide candidates A02Hexon$_{TLLY}$ or A01Penton$_{STDV}$, respectively were isolated using the IFN-γ-based cytokine secretion assay (CSA). The frequency of IFN-γ-secreting cells among CD3$^+$, CD3$^+$CD4$^+$ and CD3$^+$CD8$^+$ T cells in the CSA fractions before (“Origin”) and after enrichment (“Eluate”) were determined by multicolor flow cytometry. Representative dot plots for one strong responder to A02Hexon$_{TLLY}$ and A01Penton$_{STDV}$, respectively, show the results of qualitative analysis of IFN-γ-secreting HAdV-specific T cells [%]. The percentages of CD3$^+$IFN-γ$^+$ responses were defined in viable CD3$^+$ T cells, and the percentages of CD4$^+$IFN-γ$^+$ and CD8$^+$IFN-γ$^+$ responses were defined in viable CD4$^+$ and CD8$^+$ T cells, respectively.
